# Spectral Similarity Measures for In Vivo Human Tissue Discrimination Based on Hyperspectral Imaging

**DOI:** 10.3390/diagnostics13020195

**Published:** 2023-01-05

**Authors:** Priya Pathak, Claire Chalopin, Laura Zick, Hannes Köhler, Annekatrin Pfahl, Nada Rayes, Ines Gockel, Thomas Neumuth, Andreas Melzer, Boris Jansen-Winkeln, Marianne Maktabi

**Affiliations:** 1 Innovation Center Computer Assisted Surgery (ICCAS), Faculty of Medicine, Leipzig University, 04103 Leipzig, Germany; 2 Department of Visceral, Transplant, Thoracic, and Vascular Surgery, University Hospital of Leipzig, 04103 Leipzig, Germany; 3 Institute for Medical Science and Technology (IMSaT), University of Dundee, Dundee DD1 4HN, UK; 4 Department of General, Visceral, Thoracic, and Vascular Surgery, Klinikum St. Georg, 04129 Leipzig, Germany; 5 Department of Electrical, Mechanical and Industrial Engineering, Anhalt University of Applied Science, 06366 Köthen (Anhalt), Germany

**Keywords:** hyperspectral data, similarity measures, tissue discrimination, spectral angle mapper, gastrointestinal, thyroidectomy

## Abstract

Problem: Similarity measures are widely used as an approved method for spectral discrimination or identification with their applications in different areas of scientific research. Even though a range of works have been presented, only a few showed slightly promising results for human tissue, and these were mostly focused on pathological and non-pathological tissue classification. Methods: In this work, several spectral similarity measures on hyperspectral (HS) images of in vivo human tissue were evaluated for tissue discrimination purposes. Moreover, we introduced two new hybrid spectral measures, called SID-JM-TAN(SAM) and SID-JM-TAN(SCA). We analyzed spectral signatures obtained from 13 different human tissue types and two different materials (gauze, instruments), collected from HS images of 100 patients during surgeries. Results: The quantitative results showed the reliable performance of the different similarity measures and the proposed hybrid measures for tissue discrimination purposes. The latter produced higher discrimination values, up to 6.7 times more than the classical spectral similarity measures. Moreover, an application of the similarity measures was presented to support the annotations of the HS images. We showed that the automatic checking of tissue-annotated thyroid and colon tissues was successful in 73% and 60% of the total spectra, respectively. The hybrid measures showed the highest performance. Furthermore, the automatic labeling of wrongly annotated tissues was similar for all measures, with an accuracy of up to 90%. Conclusion: In future work, the proposed spectral similarity measures will be integrated with tools to support physicians in annotations and tissue labeling of HS images.

## 1. Introduction

Medical hyperspectral imaging (HSI) is an emerging technique in non-invasive and contactless image acquisition that supplies spatial and spectral information collected from biological tissue [1]. The 3-dimensional data are called hypercubes or hyperspectral (HS) images and supply biochemical information. The spectra are individual spectral signatures of the tissues. The medical applications of HSI are mainly in the field of tissue perfusion evaluation as well as the discrimination of different healthy and pathological tissues. Tissue perfusion evaluation was performed for wound diagnostics and monitoring [2,3,4,5] and for the early detection and examination of arterial diseases [6]. Further, HSI was evaluated to identify tumor margins [7,8,9,10,11] such as those of melanoma [12] and brain tumors [7]. Ortega et al. presented the ability of HSI to assist pathologists in the diagnosis of histological samples by discriminating between normal and tumor breast cancer cells [13]. Tissue discrimination using HSI for the identification of risk structures was investigated. Wisotzky et al. developed a hyperspectral method to analyze optical human tissue properties in vivo. Different tissue types such as fat, connective tissues, muscles, and nerves have been evaluated [14]. Barberio et al. presented the first attempt for identification, differentiation, and visualization of the parathyroid and thyroid glands, skin, connective tissue, muscle, gauze, and surgical instruments by evaluating nine patients’ HSI data [15].

Further, to help interpret generated HSI data that are sufficiently complex, artificial intelligence approaches have been used [16]. Machine learning classification methods have been evaluated to automatically identify human tumor tissue and cells in intraoperative macroscopic HSI data and microscopic HSI data of pathological slides [17,18], as well as non-pathological structures [19]. Maktabi et al. showed promising results using a support vector machine (SVM) to classify endocrine tissue [9]. Moreover, deep learning methods, such as the convolutional neural network (CNN), have been used to provide the deep spectral features of tumor tissue. Thiem et al. used a deep neural network to detect oral malignancies [19]. In addition, Grigoroiu et al. showed that CNN has the potential to provide a color-based classification approach during real-time HSI in endoscopy [20]. Cervantes-Sanchez et al. proposed an automated tissue segmentation method for liver and neck tissue using a machine learning method [21]. Barberio et al. used a CNN method for in vivo nerve detection [22], and Jansen-Winkeln et al. used CNN for automatic classification of colorectal cancer and healthy mucosa [23]. Machine learning and deep learning methods are reliable and able to provide suitable models for a versatile range of available medical HS data. These methods often require large computational expenditures and large annotated datasets to train the models.

The present study investigated spectral similarity measures that effectively employ the spectral signature information for biological tissue discrimination. Precisely, object discrimination accuracy, precision and validity rely on similarity measures and the correctly defined target signature. Earlier, researchers used spectral similarity measures for spectral signature detection in the geographical, geological, and agriculture fields. Spectral measures have been used for the identification of crops [24], minerals [25,26], and geologically sensitive areas [27]. The spectral angle mapper (SAM) and spectral information divergence (SID) were the most often used measures [26,28,29]. However, SAM is unable to distinguish between positive and negative correlations and unable to correctly match badly illuminated target pixels [30]. Du et al. developed a hybrid measure by combining SAM and SID methods with trigonometric functions [31]. Results have shown that the ability of discrimination is improved. Kumar et al. developed a hybrid method based on the spectral correlation angle (SCA) and SAM methods and applied it for the wavelength range from 400 nm to 2500 nm to classify Vigna species. Results were promising [24] but were suitable only in the spectral region 400 nm to 700 nm. Adep et al. showed that SID-SAM and SID-SCA obtained better performance for mineral classification [25].

Spectral similarity measures were also evaluated in medicine in a limited number of studies. Martin et al. used SAM for detecting altered mucosa of the human larynx [32]. Chen et al. presented the SAM method as a non-invasive method for determining and analyzing the skin changes in systemic sclerosis in humans [33]. SAM scores obtained from 31 patients were significantly higher than the normal skin score, though clinicians could not visually detect any skin abnormality. In the study by Fabelo et al., the spectral angle mapper was used to find similar spectra in an HSI cube [34].

This study aimed to analyze which similarity measures are best-suited for the discrimination of spectral data of human tissue. Several deterministic and probabilistic similarity measures were evaluated. Further, we developed two optimized hybrid spectral similarity measures as a combination of known similarity measures that are deterministic and probabilistic. We evaluated the performance of the proposed methods in two case studies, the discrimination of thyroid and colon structures from surrounding tissues and surgical instruments. Moreover, we tested the new spectral similarity measures to automatically check the identity of tissues manually labeled by users and to correct wrong annotations by comparing their spectral signatures with other available tissue signatures stored in a database. This preliminary study aimed to demonstrate the potential use of spectral similarity measures to assist surgeons and researchers in tissue annotation.

## 2. Material and Methods

### 2.1. Patient Data

The HSI data used in this paper were collected from the University of Leipzig Medical Center. This study was approved by the ethics committee of the University Hospital of Leipzig, Germany (393/16-ek). Images between 500 nm to 1000 nm were recorded by using the TIVITA^®^ Tissue system (Diaspective Vision GmbH, Am-Salzhaff-Pepelow, Germany). It is a push broom scanner HSI camera with a spectral resolution of 5 nm. The HS data cube has a dimension of 640 × 480 × 100 pixels (x-, y-, wavelength). The camera was placed at 50 cm from the in vivo tissue intraoperatively.

Data on a total of 100 patients were used. Nine patients were recorded during thyroidectomies/parathyroidectomies, and 91 during gastrointestinal surgeries. In vivo tissues and surgical material visible in the HSI data were labeled by an experienced surgeon. The annotations in the thyroid/parathyroid dataset were thyroid, parathyroid, muscle, nerve, skin, instruments, and gauze. A total of 147,799 spectral signatures were collected (Table 1).

The second gastrointestinal dataset included 138,962 spectral signatures of the small intestine, colon, fat, gallbladder, esophagus, pancreas, spleen, and stomach (Table 2). The summary of the dataset is given below.

### 2.2. Pre-Processing of HSI Data

**Figure 1:**
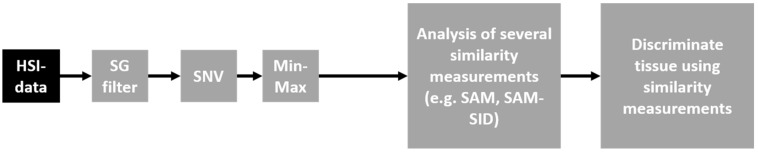
Pipeline of the overall approach.

Figure 1 depicts the different steps of our approach. A Savitzky–Golay (SG) filter with a polynomial order of three was used for spectral data smoothing. This filter eliminates the high-frequency noise. It replaces each value of the spectra with a new value that is obtained by polynomial fit without distorting it. Further, the spectral data were normalized using the standard normal variate (SNV) algorithm by adjusting the reflectance mean to 0 and standard deviation to 1. To avoid the divergence calculation error resulting from negative data values in the vector array in the dataset, it was necessary to further rescale the data to obtain positive values with the Min-Max filter.

### 2.3. Spectral Similarity Measures

Measuring the similarity between data vectors was performed in HSI applications. In this part, six classical discriminatory spectral measures were studied. These methods were suitable to evaluate the tissue’s discriminatory ability. Apart from this, a newly optimized spectral measure method was presented.

In the following, Δ = ${\left\{s_{k}\right\}}_{k=1}^{K}$ is a dataset of *K* labeled spectral signatures. Let us assume that *s_i_* and *s_j_* are spectral signatures of two pixel vectors, where *s_i_* = (*s_i1_, s_i2_, …, s_iL_*)^T^ and *s_j_* = (*s_j1_, s_j2_, …, s_jL_*)^T^. *L* is the number of wavelengths or spectral bands.

#### 2.3.1. Spectral Angle Mapper (SAM)

The SAM measure, a supervised classification method [35], was introduced in 1993 [28]. It is a deterministic approach that calculates the angle between two spectra over the *L* wavelengths. Its values are included in the range 0 to π/2 radians. The smaller angle shows higher spectral similarity.

(1) $$\mathrm{SAM}\;\left(s_{i},s_{j}{}_{\;}\right)=\cos^{-1}\left(\theta_{s_{i},s_{j}{}_{\;}}\right)$$

where the spectral angle $\theta_{s_{i},s_{j}{}_{\;}}$ is defined as

(2) $$\theta_{s_{i},s_{j}{}_{\;}}=\;\frac{\sum_{m,n=1}^{L}s_{im}s_{jn}{}_{\;}}{\sqrt{\sum_{m=1}^{L}s_{im}{\;}^{2}\sum_{n=1}^{L}s_{jn}{\;}^{2}\;}}$$

#### 2.3.2. Spectral Information Divergence (SID)

Based on divergence calculation, the SID, which is a stochastic method, computes the distance between the probability distribution produced by two spectral signatures [29]. The smaller value of divergence represents the best similarity between the vectors.

(3) $$\mathrm{SID}\;\left(s_{i},s_{j}{}_{\;}\right)=entropy\left(s_{i}||s_{j}{{}_{\;}}_{\;}\right)+entropy(s_{j}{}_{\;}||s_{i})$$

Here, $entropy\left(s_{i}||s_{j}{{}_{\;}}_{\;}\right)$ is the relative entropy of $s_{j}$ with respect to $s_{i}$, and $entropy\left(s_{j}{}_{\;}||s_{i}\right)$ is the relative entropy of $s_{i}$ with respect to $s_{j}$. The entropy is defined as the probability distribution of both spectra. The relative entropies are computed using the Kullback–Leibler information divergence [36], which is widely used in information theory.

#### 2.3.3. SAM-SID Mixed Measures

SAM computes angle and gives the correlation between two spectra. SID calculates divergence in terms of distance. Du et al. presented a hybrid method [31] by combining these two methods in two different combinations,

(4) $$\mathrm{SID}-\mathrm{SIN}\left(\mathrm{SAM}\right)=\;\mathrm{SID}\;\times sin\left(\mathrm{SAM}\right)\;;\;\mathrm{SID}-\mathrm{TAN}\left(\mathrm{SAM}\right)=\;\mathrm{SID}\times tan\left(\mathrm{SAM}\right).$$

where *sin* and *tan* are the usual sine and tangent trigonometric functions. The *tan* version showed superior results in comparison to the *sin* version.

The second variant is noted as SID-TAN(SAM) in the following.

#### 2.3.4. Spectral Correlation Angle (SCA)

The SCA is based on the Pearson correlation coefficient. It is similar to the SAM method. SCA eliminates the negative correlation and enhances the better shading effect in comparison to SAM [30,37,38]. The SCA values range between 0 and π/2 radians.

(5) $$\mathrm{SCA}\left(s_{i},s_{j}{}_{\;}\right)=\cos^{-1}\left[\left(\sigma \left(s_{i},s_{j}{}_{\;}\right)+1\right)/2\right]$$

where $\sigma \left(s_{i},s_{j}{}_{\;}\right)$ is the Pearson correlation coefficient.

The Pearson correlation coefficient is defined as

(6) $$\sigma \left(s_{i},s_{j}{}_{\;}\right)=\frac{L\sum_{m,n=1}^{L}s_{im}s_{jn\;}-\sum_{m=1}^{L}s_{im}\sum_{n=1}^{L}s_{jn\;}\;}{\sqrt{\left[L\sum_{m=1}^{L}s_{im}{\;}^{2}-\sum_{n=1}^{L}s_{in}{\;}^{2}\;\right]}[L\sum_{m=1}^{L}s_{jm}{\;}^{2}-\sum_{n=1}^{L}s_{jn}{\;}^{2}\;]}$$

The values of the Pearson correlation coefficient are included in the range −1 and +1.

#### 2.3.5. SID-SCA Mixed Measures

Kumar et al. proposed two SID-SCA mixed measure methods [24], which are calculated in the following manner:

(7) $$\mathrm{SID}-\mathrm{SIN}(\mathrm{SCA})=\mathrm{SID}\times \sin\left(\mathrm{SCA}\right);\;\mathrm{SID}-\mathrm{TAN}(\mathrm{SCA})=\mathrm{SID}\times \tan\left(\mathrm{SCA}\right).$$

The second variant is noted SID-TAN(SCA) in the following.

#### 2.3.6. Bhattacharyya Distance-Based Jeffries–Matusita Measures (JM)

The Bhattacharya distance or coefficient BC measures how similar two probability distributions are. It is widely used to calculate the separability between two different spectra. It is calculated as follows:

(8) $$\mathrm{BC}\left(s_{i,},\;s_{j\;}\right)=\sum_{m,n=1}^{L}\sqrt{s_{im}s_{jn\;}}$$

In the literature, the JM distance is an enhancement over the BC and scales the distance between 0 and 2 [39].

(9) $$\;\mathrm{JM}=\sqrt{2\left(1-e^{-\mathrm{BC}\left(s_{i,},\;s_{j\;}\right)}\right)}$$

#### 2.3.7. New Optimized Hybrid Measures (SID-JM-TAN(SCA))

Adep et al. showed that the combination SID-TAN(SCA) has a higher ability than the SID-SIN(SCA) to identify the target spectra for mineral classification [25]. The reason is that the *tan* function calculates the perpendicular distance between the spectra *t* and s. The JM distance is used for separability criteria and is optimal for classification tasks [27]. The hybrid JM-SAM method was presented and resulted in a higher spectral discriminability in comparison to SAM and JM using landcover HS images.

In this work, a new hybrid measure based on the tangent function was developed by combining a deterministic method, i.e., SCA and SAM, and two stochastic methods, i.e., SID and JM. Combination of these measures increases the chance of detecting the similarity and dissimilarity between spectra.

(10) $$\mathrm{SID}-\mathrm{JM}-\mathrm{TAN}\left(\mathrm{SCA}\right)=\mathrm{SID}\times \mathrm{JM}\times tan\left(\mathrm{SCA}\right);\;\;\mathrm{SID}-\mathrm{JM}-\mathrm{TAN}\left(\mathrm{SAM}\right)=\mathrm{SID}\times \mathrm{JM}\times tan\left(\mathrm{SAM}\right)$$

Both measures are noted SID-JM-TAN(SCA) and SID-JM-TAN(SAM) in the following.

### 2.4. Statistical Analysis for Comparing Spectral Similarity Measures 

The spectral similarity measures are given with different units. Therefore, Naresh Kumar et al. introduced three statistical methods in order to be able to compare and evaluate them [24]. They are described below.

#### 2.4.1. Relative Spectral Discriminatory Power (RSDPW)

Let us assume that *t* is any specific target spectrum that is compared with the spectra $s_{i}$ and $s_{j}$. *m*(.,.) is any spectral similarity measure previously defined. The RSDPW is defined as follows:

(11) $$\mathrm{RSDPW}\;(s_{i},s_{j};t)=m\mathrm{ax}\left\{\begin{array}{c} \frac{m\left(s_{i}\;,\;\;t\right)}{m\left(s_{j}\;,\;t\right)},\frac{m\left(s_{j}\;,\;t\right)}{m\left(s_{i}\;,\;t\right)} \end{array}\right\}$$

The high values of RSDPW ($s_{i},s_{j};t)$ show the better discriminatory power of the analyzed measures.

#### 2.4.2. Relative Spectral Discriminatory Probability (RSDPB)

Δ = ${\left\{s_{k}\right\}}_{k=1}^{K}$ is a dataset of *K* labeled spectral signatures. The RSDPB of all *s_k_*‘ s in Δ relative to *t* is defined as follows:

(12) $$RSDPB\;\left(P_{t,\;\Delta \;}\left(k\right)\right)=m\left(t,\;s_{k}\right)/\sum_{i=1}^{K}m\left(t,\;s_{i}\right)$$

$P_{t,\;\Delta \;}$ is the relative spectral discriminatory probability vector of Δ with respect to *t*. $\sum_{i=1}^{K}m\left(t,\;s_{i}\right)$ is a normalized value determined by *t* and Δ.

The RSDPB provides a relative spectral similarity measure and, therefore, enables a more accurate comparison between different tissues. If the RSDPB is low, the spectral similarity method has high applicability.

#### 2.4.3. Relative Spectral Discriminatory Entropy (RSDE)

Further, the RSDE of the spectral signature t with respect to the dataset Δ is defined as

(13) $$\mathrm{RSDE}\;\left(t;\Delta \right)=-\sum_{k=1}^{K}(P_{t,\;\Delta \;}\left(k\right)\;\log\left(P_{t,\;\Delta \;}\left(k\right)\right)$$

Small values of RSDE specify the superiority of the method.

### 2.5. Quantitative Evaluation of All Similarity Measures

**Figure 2:**
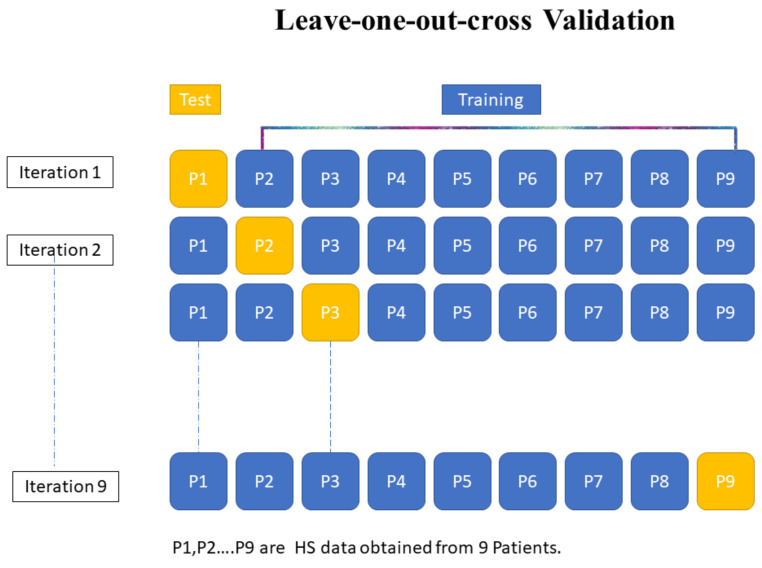
Systematic representation of the leave-one-patient-out cross-validation methodology based on the HS data of 9 patients.

For quantitative evaluation, the spectral similarity measures were tested using the two patient databases. Their performances were evaluated using the three statistical methods described previously. These experiments were conducted using a leave-one-patient-out cross-validation (LOPOCV) method (Figure 2). We considered a dataset including the tissue spectra of N patients. We selected the thyroid and colon as target tissues. For each evaluation step, the target tissue spectra of one patient were tested, and the spectra of the N-1 other patients were used as reference spectra. This operation was repeated N times, so that each patient was tested independently. The mean spectra of a given tissue for each patient are considered in the calculation of the spectral similarity measures.

### 2.6. Application of Similarity Measures to Support Tissue Annotation

An application to automatically support tissue annotations performed by users and using the spectral similarity measures is presented. Firstly, the label of a target spectrum *t* previously annotated by medical experts is checked. Secondly, a new label is automatically suggested for the annotated target spectrum *t*, identified as incorrect. Both steps compare the target spectrum with reference spectra whose labels, e.g., *tissues A*, *B* and *C*, are known, using the spectral similarity measures. In order to speed up the process, the target spectrum is not compared with all reference spectra, but with the centers of clusters of spectra similarly labeled, obtained using the K-means clustering with two clusters.

In the first step, a threshold-based check was performed. The thresholds T*_tissueA, m_(r)* were calculated as the mean values of the spectral similarity measures *m* between the cluster centers *r* and all other spectra labeled *tissue A* within the cluster. Afterwards, these thresholds T*_tissueA, m_(r)* were used to check if a target spectrum *t* labeled *tissue A* was correct. If the spectral similarity values between *t* and all other cluster centers labeled *tissue A* were lower than the thresholds T*_tissueA, m_(r)*, the label *tissue A* of the target spectrum *t* was assumed to be correct. The spectra labeled thyroid and colon were checked. A LOPOCV on both datasets separately was conducted to evaluate this approach.

The second steps suggested a tissue label for the target spectrum whose label was identified as incorrect. The suggested label corresponded to the cluster with the smallest spectral similarity measure value. For example, the target spectrum *t* was compared with three clusters with labels *tissue A*, *tissue B* and *tissue C*. So, three spectral similarity values were calculated. The label of the cluster with the smallest value was chosen as correct. The predicted tissue label was compared with the annotation of *t* performed manually by the physician, which was assumed to be correct. This was conducted for each biological tissue in both databases and similarity measures. For the thyroid/parathyroid dataset, a LOPOCV was performed. For the gastrointestinal dataset, a k-fold cross validation using k= 10 was performed. The data were split according to patients, whereby the same patient would not appear in two different folds. The folds had the same percentage of samples for each class.

The performance of both steps was estimated using the accuracy. It was the proportion of true positives and true negatives in all evaluated predictions.

## 3. Results

The spectral similarity measures SAM, SID, SID-TAN(SAM), SID-TAN(SCA), SID-JM-TAN(SAM) and SID-JM-TAN(SCA) were evaluated statistically using the two different datasets described in Section 2.1. The similarity measures were developed and evaluated statistically using Python (version 3.7) and the pysptools library (version 0.15.0).

### 3.1. Study 1 (In Vivo Thyroid/Parathyroid Dataset)

**Figure 3:**
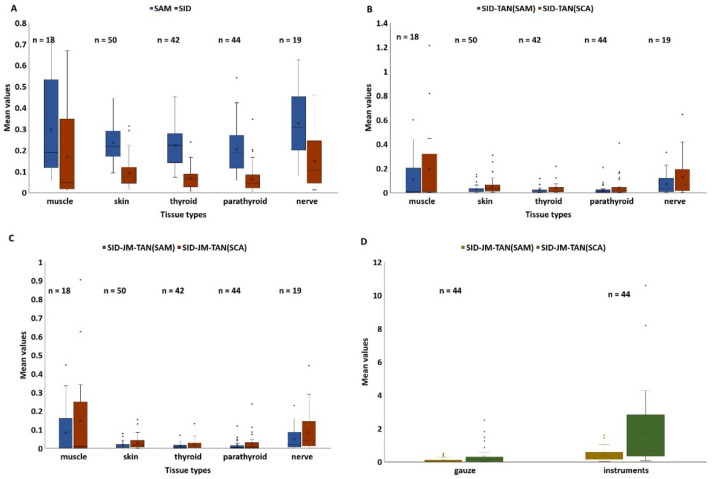
Averaged similarity values calculated for thyroid as reference versus muscle, nerve, parathyroid, thyroid, gauze, and instruments ((**A**) SAM and SID measures; (**B**) SID-TAN(SAM) and SID-TAN(SCA) measures; (**C**) SID-JM-TAN(SAM) and SID-JM-TAN(SCA) measures; (**D**) SID-JM-TAN(SAM) and SID-JM-TAN(SCA) for gauze and instruments against thyroid); n is the number of spectra.

In the first study, the thyroid tissue was selected as a reference and compared with other human tissues, i.e., muscle, nerve, skin, parathyroid, surgical instruments and gauze. The results of the computation of the spectral similarity measures are represented in Figure 3. Gauze and instrument spectral data produce much higher similarity values in comparison to other tissue spectral data (Figure 3D).

SID-TAN(SCA) and SID-JM-TAN(SCA) showed the lowest similarity values for thyroid versus parathyroid and thyroid versus skin in comparison to the other similarity measures. The similarity value measured by SAM between thyroid versus thyroid and thyroid versus parathyroid was very close; indeed, a remarkable difference was visible while comparing skin, parathyroid, muscle, and nerve. On the other hand, the averaged similarity value calculated by SID-JM-TAN(SCA) between thyroid versus thyroid was 1.1 times smaller than that between thyroid versus parathyroid. For dissimilar spectra, SID-JM-TAN(SCA) produced the most significant value compared to the other five measures. The SID-JM-TAN(SCA) method measured up to 6.7 times higher values for dissimilar spectra. Contrarily, SAM measured only up to 1.5 times higher values for dissimilar spectra. So, based on the smallest produced value, SID-JM-TAN(SCA) and SID-JM-TAN(SAM) measures are both highly recommended. Most of the methods can analyze the similarity found between two spectral signatures.

**Figure 4:**
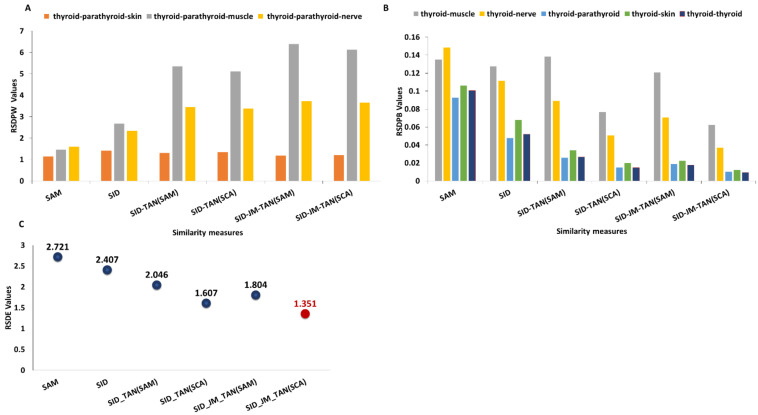
(**A**) Relative spectral probability (RSDPW) for different similarity measures. The thyroid was selected as a target and compared to other tissues. (**B**) RSDPB value produced by different measures (target tissue: thyroid). (**C**) RSDE values produced by different similarity measures (target tissue: thyroid).

Figure 4A shows the RSDPW values calculated between two similar tissues, thyroid and parathyroid, and a third one. The RSDPW values obtained with SID-JM-TAN(SAM) and SID-JM-TAN (SCA) were relatively higher than the RSDPW values obtained with other measures. A higher value of RSDPW shows the better discriminatory power of a measure. Comparing RSDPW_SID-JM-TAN(SCA)(thyroid-parathyroid-nerve)_ = 3.66, RSDPW_SID-JM-TAN(SCA) (thyroid-parathyroid-muscle)_ = 6.12, and RSDPW_SID-JM-TAN(SCA) (thyroid- parathyroid-skin)_ = 1.21 implies that the signatures of nerve, muscle, and skin are dissimilar. The results indicate that the SID-JM-TAN(SAM) and SID-JM-TAN(SCA) measures are suitable for tissue discrimination.

Figure 4B shows the RSDPB values that represent the relative capability of all spectra to be discriminated from others and, therefore, evaluate the performance of different spectral measure methods. It was quite noticeable that SID-JM-TAN(SCA) showed the lowest values for muscle, nerve, parathyroid, and skin, and a relatively smaller value for thyroid, which was our reference tissue. Lower RSDPB values showed that the target spectra matched the spectra in the dataset. SID-JM-TAN(SCA) showed a 9-fold smaller relative difference than the SAM for discriminating parathyroid against thyroid tissue. SID-JM-TAN(SCA) showed a 8.5-fold smaller relative difference than SAM for discriminating skin against the thyroid. So, SID-JM-TAN(SCA) is highly suitable for tissue classification.

Figure 4C shows the relative spectral discriminatory entropy. The RSDE calculated for the SID-JM-TAN(SCA) was lower than for the other analyzed measures.

### 3.2. Study 2 (In Vivo Gastrointestinal Surgery Dataset)

In the second case study, the colon tissue was used as a reference and compared to the tissue of other gastrointestinal organs.

**Figure 5:**
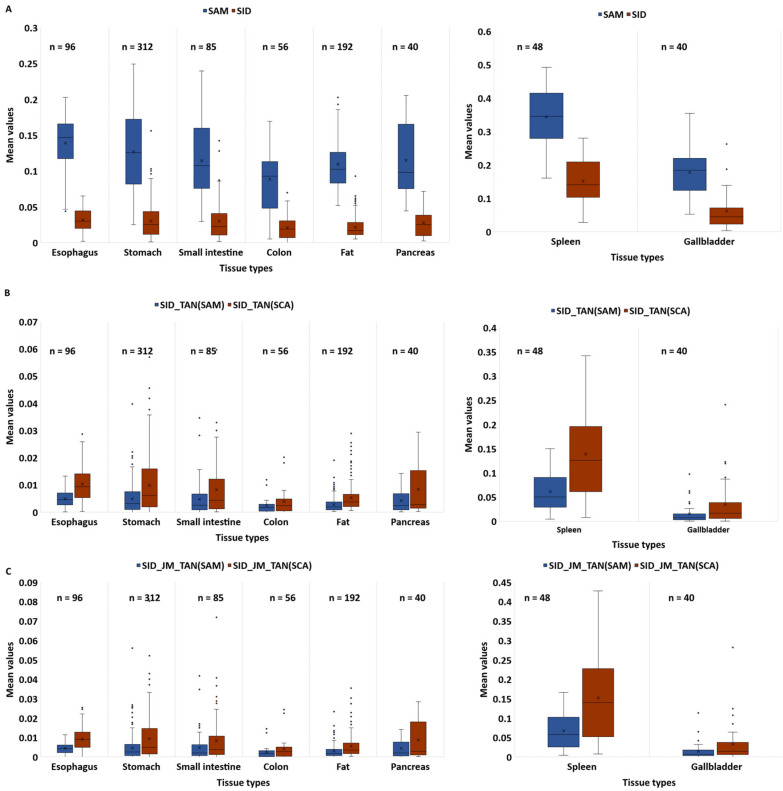
Averaged similarity values calculated by different methods to compare colon tissue with other tissues (e.g., esophagus, stomach, small intestine, colon, fat, pancreas, gallbladder, and spleen) ((**A**) SAM and SID measures; (**B**) SID-TAN(SAM) and SID-TAN(SCA); (**C**) SID-JM-TAN(SAM) and SID-JM-TAN(SCA)).

Figure 5 compares the similarity values calculated by different similarity measures in between available spectral signatures in the database and for colon. The similarity values for spleen and gallbladder signatures against colon were significantly higher and remarkable. Further, SAM, SID, SID-TAN(SAM), SID-TAN(SCA), and SID-JM-TAN(SAM) were capable of discriminating colon signature against other available spectra in the database. However, our proposed method, SID-JM-TAN(SCA), showed better performance, due to the higher relative differences between tissues. For example, in the SAM method, the relative difference was 1.5 times larger for the esophagus against colon than for colon against colon. However, the SID-JM-TAN(SCA) method showed a relative difference that was 2.5 times larger.

**Figure 6:**
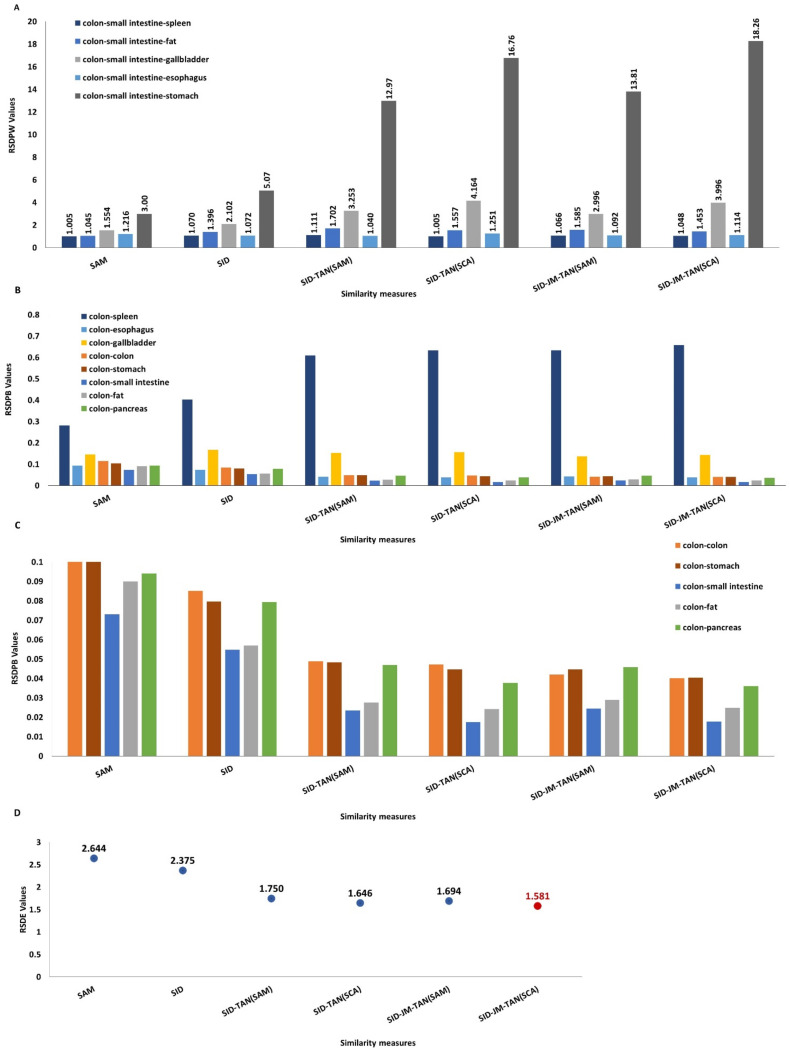
(**A**) RSDPW produced by different similarity measures. (**B**) RSDPB values calculated by different similarity measures. (**C**) RSDPB values produced by the small intestine, colon, fat, pancreas, and stomach. (**D**) RSDE values produced by different similarity measures (reference tissue: colon).

Figure 6A shows the RSDPW values computed between two similar tissues, colon and small intestine, and a third one. A higher value of RSDPW shows the better discriminatory power of a measure. For any target signature, all of the above-mentioned measures are suitable for discriminating other tissue signatures relative to the chosen target tissue. RSDPW_SID-JM-TAN(SCA) (colon-small intestine-spleen)_ = 18, RSDPW_SID-JM-TAN (SCA) (colon-small intestine-gallbladder)_ = 4 and RSDPW_SID-JM-TAN (SCA) (colon-small intestine-esophagus)_ = 1.1 suggested that the signature of the spleen was quite different from that of small intestine relative to gallbladder and esophagus. RSDPW _(colon-small intestine-stomach)_ showed all values between 1 and 2, representing the similar nature of small intestine and stomach tissue. Regarding the evaluation of the measures, RSDPW_SID-JM-TAN (SCA)_ showed relatively higher values in comparison to SAM. This evidence also suggests that the combination of stochastic and deterministic measures is more powerful than the individual measures.

Figure 6B,C represent the RSDPB values corresponding to the discriminatory capability of the similarity measures. From the plots, it was assumed that the smaller value of RSDPB represents the closeness of a reference to the target. Higher values of the RSDPB were obtained between colon and spleen.

The relative spectral discriminatory entropy RSDE measured by SID-JM-TAN(SCA) was lower when compared to other measures (Figure 6D). A smaller entropy value means the target tissue spectra are correctly matched. Therefore, both measures proved to be a better in discriminating tissue spectra.

### 3.3. Application to Support Tissue Annotation

We evaluated the approach to automatically support the annotations of tissues. The first step used thresholds on the spectral similarity measures to check the tissue labels. For the thyroid/parathyroid dataset, both hybrid measures showed the best results. The SID-JM-TAN(SCA) correctly identified thyroid tissue in three out of seven patients. In failure cases, thyroid was detected as parathyroid or skin. For the gastrointestinal dataset, the hybrid measure SID-JM-TAN(SAM) showed the best results. It showed that in four out of eight patients, the colon was correctly annotated. Only the hybrid measure SID-JM-TAN(SCA) showed similar results with less accuracy. In the end, the similarity measures could correctly check the annotations of the spectra in 73% of the total spectra labeled thyroid and in 60% of the total spectra labeled colon. The hybrid similarity measures showed higher performance.

In the second step, the tissue label corresponding to the reference spectra cluster with the smallest spectral similarity measure value was suggested for the target spectrum identified as incorrectly annotated in the previous step. The thyroid/parathyroid dataset was evaluated in a binary classification, where only thyroid and parathyroid tissues were labeled. The accuracies were similar for all spectral similarity measures and equal to 76%. The accuracies obtained on the gastrointestinal dataset were similar for all similarity measures for all tissues, too. An accuracy of 78% for esophagus, 64% for intestine, 69% for fat, 66% for pancreas, 77% for colon, 53% for stomach, 85% for gallbladder, and 90% for spleen was obtained. The results showed that automatic tissue labeling using spectral similarity measures is feasible with acceptable accuracy.

## 4. Discussion

In this work, we developed two new hybrid spectral similarity measures to discriminate tissues. They combine SID, JM, and tan of SAM and SCA, respectively. We applied them on HS images with thyroid/parathyroid and gastrointestinal tissues in a spectral range from 540 nm to 995 nm. Our developed hybrid measures SID-JM-TAN(SAM) and SID-JM-TAN(SCA) demonstrated their potential to identify the tissues. They produced higher discrimination values, by up to 6.7 fold, than the other classical spectral similarity measures, to discriminate dissimilar tissues. The RSDE values of the hybrid measures (RSDE_SID-JM-TAN (SCA)_= 1.58 for colon) showed better applicability than standard similarity measures such as SAM (RSDE_SAM_= 2.64 for colon).

The correct annotation of HS images with anatomical labels corresponding to tissues is a necessary step for further automatic classification tasks. Therefore, an application of the similarity measures to automatically support experts with the task of tissue labeling was presented. Few works used the similarity measures for this task [33,35]. Furthermore, they used only SAM to compare spectral signatures. In this study, we suggested different similarity measures. In the first step for label checking, the correctness of tissue annotations was established for 73% of the total spectra labeled thyroid and for 60% of the total spectra labeled colon. The hybrid measures showed the best results. In the second step, which suggested a label for a spectrum identified as wrongly annotated by the user, the task was successfully performed with averaged accuracies of 76% for the thyroid/parathyroid dataset and 83% for the gastrointestinal dataset. Although the approach still needs to be improved, we were able to demonstrate that the similarity measures are simple tools to support the annotation of HS images. A great benefit of the spectral similarity measures over machine learning approaches is that no further training is necessary. This automatic tool is crucial to obtain high-quality datasets for artificial intelligence algorithms. Moreover, manual data annotation is a very time-consuming and challenging task.

A limitation of our study was the limited number of thyroidectomy data. In further studies, more data should be involved.

Similarity measures are highly dependent upon the number of samples and the quality of data available. We can achieve more accurate spectral signatures in the spectral library with respect to their specific classes by using this method. In future works, the capability of spectral measurements to discriminate malign and healthy tissue should be analyzed. However, the available HSI data are sufficient for preliminary study to investigate and evaluate the measures.

## 5. Conclusions

The work presented in this paper evaluated spectral distance measures for the characterization of tissues using HS imaging data. The enormous amount of spectral information obtained from hyperspectral imaging is suitable for tissue detection. A comparative study among six measures, SAM, SID, SID-TAN(SAM), SID-TAN(SCA), SID-JM-TAN(SAM), and SID-JM-TAN(SCA), was performed.

Our newly improved hybrid measures, SID-JM-TAN(SAM) and SID-JM-TAN(SCA), were shown to be the most suitable measures that can be used for tissue characterization.

We showed that spectral similarity measures have the potential to automatically support tissue annotation tasks. It is worth noting that our proposed hybrid spectral similarity measures delivered good accuracy.

## Figures and Tables

**Table 1:** Spectral data of the thyroid/parathyroid dataset.

Patient	Instrument	Gauze	Thyroid	Parathyroid	Skin	Muscle	Nerve
1	6285	-	10,853	-	8078	1450	197
2	1257	3778	3625	-	5525	1257	-
3	-	2514	12,570	2774	11,102	-	-
4	2514	1257	1257	1257	3209	-	-
5	5028	-	3847	441	8346	1839	-
6	5825	3771	-	441	1257	-	-
7	3396	5028	-	882	2514	-	226
8	-	6292	1257	317	-	-	831
9	441	3771	9124	882	1264	-	-
Frequency	24,746	26,411	42,533	6994	41,295	4546	1254
In Percent (%)	16.75	17.87	28.8	4.73	27.94	3.07	0.84

**Table 2:** Spectral data of the gastrointestinal dataset.

No. of Participating Patients	Tissue Type	Spectral Signatures	Percentage (%)
11	Small Intestine	15,913	11.45
7	Colon	8897	6.40
24	Fat	24,912	17.92
5	Gallbladder	11,475	8.25
12	Esophagus	12,272	8.83
5	Pancreas	8799	6.33
6	Spleen	7542	5.43
39	Stomach	49,552	35.65
Total: 91		138,962	100

## Data Availability

The data presented in this study can be obtained upon request at the following email: claire.chalopin@medizin.uni-leipzig.de.
